# Costly Sacrifices

**Published:** 1891-05-16

**Authors:** 


### The Doctor's Armchair.

### COSTLY SACRIFICES.

The Archbishop of York has died of a chest affection. His death was premature, though he was seventy. He had five or six years of good work left in him if an untimely accident had not cut short his life. A great many other men, less eminent than the Archbishop, but useful to the world, and indispensable to their families, have also died of chest affections during the last few weeks; some of them hardly beyond their prime. Savages, untutored children of nature, have always been accustomed, and still are accustomed, to regard such things as inevitable; they acquiesce in them, meekly or defiantly according to their temper; but they do acquiesce. They feel that they cannot put forth any intelligent effort at prevention.

The bolder spirits of this tutored age do not hesitate to question many of the seeming certainties and everlasting necessities of past times. The questioning spirit is a wholesome spirit, provided it be reverent and reasonable. Questioning that is merely impertinent ought to be whipped. But there is no doubt of two things: first, that the eternal law-giver intended man to have a large dominion over nature; and, secondly, that man has by no means, as yet, attained to the full extent of the conquests which are possible for him. We have not hesitated in these pages to speak with great plainness of certain failures in the region of scientific medical inquiry: still less do we hesitate to apply both whip and spur to true medical scientists in order to urge them forward with accelerated speed on the pathway of sure advance.

The death of the Archbishop of York and of so many other valuable persons, the widespread epidemic of the peculiar disease called, for want of a better name, "Influenza," and the long disablement of tens of thousands of unknown but useful men and women during the past few weeks, cannot but give a medical man sudden and serious pause; and cannot but make him question himself, question his art, and question his environment with deep and even painful earnestness. For it is a heavy loss to the intellectual and moral forces of the world when the lives of invaluable public men like Dr. Magee are diminished by a sixth of their working time; and it is a still more grievous injury to private families when their bread-winners are suddenly taken away before any sufficient provision has been made for the future wants of the household. Such calamities must be taken to heart by people who profess to be both scientific and practical. We will try to take them to heart in a plain and business-like way with our readers.

Now this general statement is true—that no man or woman ever dies without a reason. Nothing ever happens without a reason; and, as the greater includes the less, it is obvious, even to non-medical persons that no man or woman can die without a reason. But that which is clear to the layman as a matter of logic is urgently impressed upon the medical man as a matter of experience. He sees constantly that people die from some ascertainable cause or causes; he sees moreover that those causes are sometimes in the people themselves, sometimes in their surroundings or environment, and sometimes in both. But he sees still further—that many of the causes which produce death, both causes in the individual and causes in his environment, are manageable and controllable. An intelligent physician could many a time save life if he were called in time, and if he were allowed an absolutely free hand, unfettered by questions of expense or other considerations.

It is desirable, however, to go a step further than the early calling in of the physician, and the allowing him of a free hand. We must ask what the intelligent layman can do himself to control the causes of death which are partly personal to him and partly prevalent in his environment? Is it inevitable that educated men and women must go on for ever destitute of such elementary practical knowledge of themselves and their surroundings as would, if used at the right time, bar the door against death when he approached it with a premature knock? It is not inevitable. A few men both can and do keep death at a distance by a peculiar kind of knowledge and skill such as ordinary persons do not possess. There is a very eminent physician now living and in full work who has had a bowing acquaintance with death for many years. That physician's life is still going on, solely because in the innings the batting has been better than the bowling.

But is it the fact that middle-aged men and women play a game with death which is in some respects comparable with cricket, and in which the human creature is the batsman and death is the bowler? It is the fact, and more especially in the opening season of the year, when we have winter one day, summer the next, and autumn on the third. It may be taken as generally true that every man and woman who dies of pneumonia or acute bronchitis under sixty-eight years of age is the victim either of bad luck or of bad management. But what, then, is to be done? Men cannot be in daily consultation with their doctors throughout the spring months. It is not necessary that they be in daily consultation with their doctors, but it is necessary that they be in daily consultation with themselves. Nobody but a blockhead will deny that it is as important to attend to the daily business of keeping one's self alive as to the daily business of bringing out a satisfactory balance-sheet at the end of the year.

What is the one great health problem which requires to be solved in the English spring? Is it not this and no other—to equalise the unequal temperature? Of course unthinking people will say that this is impossible: but it is not impossible. By a careful study of the wind, an intelligent graduation of the home and office fires, and a wardrobe so elastic as to give us every variety of clothing at a moment's notice it is not impossible—when all these conditions are fulfilled—to be completely armed and equipped against a dozen changes of weather in a day. If in addition to this intelligent watchfulness and preparedness against a treacherous environment we maintain a good level of personal appetite, exercise, sleep, and regularity in work and food, we may bid defiance to our spring climate and all its malignant works.

We have written somewhat frequently on topics of this kind during both the present and several past spring seasons; and we purpose writing upon them again in the future. To our thinking an intelligent understanding of, and preparation for, dangerous climatic conditions is of the very first importance both to the medical profession and to the public; of ten times more importance, indeed, than the recent performances of Koch. He who wins a battle is not worthy of nearly so much honour as he who prevents a war. The highest, and the most difficult task which the medical profession has to perform is the prevention of disease, not its cure. He is a great doctor who cures quickly and well; but he is a much greater who teaches people so to deal with themselves and their environment that they do not need to be cured. Many a costly human sacrifice has been offered up on the altar of physiological ignorance, carelessness, and unbelief. The time has fully come when the priesthood of medicine should enter upon a generous crusade against scientific infidels and heretics of all kinds.

